# Optimizing Delivery of Therapeutic Growth Factors for Bone and Cartilage Regeneration

**DOI:** 10.3390/gels9050377

**Published:** 2023-05-03

**Authors:** Eri Takematsu, Matthew Murphy, Sophia Hou, Holly Steininger, Alina Alam, Thomas H. Ambrosi, Charles K. F. Chan

**Affiliations:** 1 Department of Surgery, Stanford Medicine, Stanford, CA 94305, USA; 2 Blond McIndoe Laboratories, School of Biological Science, Faculty of Biology, Medicine and Health, University of Manchester, Manchester M13 9PR, UK; 3 School of Medicine, University of California, San Francisco, CA 94143, USA; 4 Department of Orthopaedic Surgery, University of California, Davis, CA 95817, USA; 5 Institute for Stem Cell Biology and Regenerative Medicine, Stanford Medicine, Stanford, CA 94305, USA

**Keywords:** therapeutic growth factor delivery, osteoarthritis, osteoporosis, controlled delivery, osteoimmunology, biomaterials

## Abstract

Bone- and cartilage-related diseases, such as osteoporosis and osteoarthritis, affect millions of people worldwide, impairing their quality of life and increasing mortality. Osteoporosis significantly increases the bone fracture risk of the spine, hip, and wrist. For successful fracture treatment and to facilitate proper healing in the most complicated cases, one of the most promising methods is to deliver a therapeutic protein to accelerate bone regeneration. Similarly, in the setting of osteoarthritis, where degraded cartilage does not regenerate, therapeutic proteins hold great promise to promote new cartilage formation. For both osteoporosis and osteoarthritis treatments, targeted delivery of therapeutic growth factors, with the aid of hydrogels, to bone and cartilage is a key to advance the field of regenerative medicine. In this review article, we propose five important aspects of therapeutic growth factor delivery for bone and cartilage regeneration: (1) protection of protein growth factors from physical and enzymatic degradation, (2) targeted growth factor delivery, (3) controlling GF release kinetics, (4) long-term stability of regenerated tissues, and (5) osteoimmunomodulatory effects of therapeutic growth factors and carriers/scaffolds.

## 1. Introduction

Osteoporosis (OP) and osteoarthritis (OA) are two major health concerns affecting millions of people worldwide, causing significant medical and financial burden [1,2]. In the United States, over 50 million individuals display low bone mass, with 10 million suffering from osteoporosis, which leads to fragility fractures that impose a yearly economic burden of USD 17–20 billion on healthcare [3,4]. Cartilage degeneration in osteoarthritis afflicts 15% of the adult population, with a lifetime risk of 40% and a societal cost estimated to be as high as 2.5% of the gross domestic product annually [5,6,7]. Therefore, there is a great and growing unmet need for novel types of regenerative medicine that could address these issues.

There exist two main approaches to treating OP, namely, inhibiting further bone resorption and promoting new bone formation. Bisphosphonates, the monoclonal antibody denosumab, and selective estrogen receptor modulators are among the current medications used to inhibit bone resorption by blocking osteoclast activity [8]. Alternatively, promoting new bone formation is achieved using parathyroid hormone (PTH) and romosozumab, a sclerostin inhibitor that reduces osteoblastic bone formation by inhibiting canonical Wnt/β-catenin signaling. Although these treatments are promising, several challenges remain. For instance, the anabolic effects of PTH are not sustained after therapy discontinuation, and hypercalcemia is among the side effects reported, possibly due to the off-target effects of PTH [9]. Thus, developing a continuous releasing system with a biomaterial may help improve the treatment.

The primary approach to managing OA revolves around mitigating pain by means of oral or injected analgesics or anti-inflammatory agents. In conjunction with these pharmacological interventions, physical therapy is employed to fortify the musculature surrounding the affected joint, thereby diminishing discomfort. In extreme cases, the utilization of orthopedic prostheses for joint or bone replacement is considered as a last resort; nevertheless, the invasive nature of these surgical procedures precludes their use in certain patients [10,11].

Targeted delivery of therapeutic growth factors (GF) using hydrogel scaffolds presents a promising avenue for addressing the current limitations of treatments for OP and OA. Examples for such therapeutic GFs for bone and cartilage regeneration include, but are not limited to basic fibroblast growth factor (bFGF), bone morphogenetic proteins (BMPs), and platelet-derived growth factor (PDGF), and their mechanism of action is well-summarized in [12]. The potential of GFs to regenerate articular cartilage makes them a promising cure for OA, while their targeted delivery allows for increased specificity and reduced side effects compared to small molecules, such as PTH [13,14]. Moreover, therapeutic GFs may receive faster approval from the FDA than small-molecule drugs, making them an attractive option for the pharmaceutical industry [15]. With all these advantages, GF therapy is a very powerful tool to regenerate weakened bone and lost cartilage.

However, GFs also possess some disadvantages, such as instability, acting on untargeted tissue, difficulty in controlling release kinetics, and unknown immunomodulatory effects. To overcome these obstacles, numerous studies have been conducted to improve GF therapy. In this review, we investigate five important considerations for the development of therapeutic protein delivery systems with the aid of hydrogels for bone and cartilage regeneration, including (1) protein protection, (2) target delivery, (3) controlled release, (4) long-term stability of regenerated tissues, and (5) osteoimmunology.

## 2. Protection from Physical and Enzymatic Degradation

GFs are potent, yet sensitive therapeutic compounds. They reach their best potential when maintained in an environment where the temperature, pH, and osmolarity suit the protein. For example, BMP-2, one of the most potent GFs to induce bone, promotes 1.7 times higher mineralization in vivo if implanted with an acidic scaffold (pH 4.8) compared to a neutral scaffold (pH 6.2) [16]. Inappropriate environments lead to hydrolysis, oxidation, isomerization, reduction, deamidation, photodegradation, and disulfide scrambling, all of which result in misfolded proteins with decreased functional activity [17]. Therefore, these physical/chemical parameters are crucial to consider when delivering proteins of interest. Besides the physical/chemical factors, it is important to be mindful of enzymatic factors that affect protein degradation. Examples include trypsin, pepsin, chymotrypsin, carboxypeptidase, and elastase [18]. In addition to considering these environmental factors, designing a scaffold/carrier for GF or engineering GF itself to reduce enzymatic degradation are essential aspects in protein delivery.

### 2.1. Usage of Carriers to Protect Growth Factors

One method to prevent therapeutic GFs from physical/enzymatic degradation is to use carriers/hydrogels to encapsulate the GFs. Liposomes, such as glycosylated liposomes, prevent GFs from enzymatic degradation [19,20] because glycosylation stabilizes lipid vesicles against further destabilization. In fact, the stability of insulin was well preserved in a glycosylated liposome even under extreme stress conditions [20]. Another example is PODS™ (polyhedrin delivery system). This product is highly durable and crystalline, allowing it to encase therapeutic GFs within a polyhedrin protein frame to protect them from degradation. A study showed significantly improved bone formation with BMP-2 encapsulated in PODS in a rat calvarial bone defect model [21]. Another study demonstrated that heparinized chitosan hydrogel as a scaffold successfully stabilized the bioactivity of BMP-2 and protected it from proteolysis [22]. In addition to its protective role of GFs, chitosan also promotes the differentiation of human mesenchymal stem cells (MSCs), showing higher mineral deposition and calcium content together with genes associated with calcium-binding and mineralization [23], serving as an attractive carrier for delivering GFs for bone/cartilage regeneration.

### 2.2. Engineering Proteins to Increase Stability of GFs

**Figure 1:**
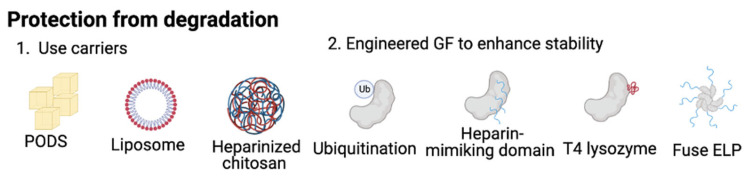
Methods to protect GFs from degradation. (**1**) Use carriers, such as PODS, liposomes, and heparinized chitosan. (**2**) Engineered GF to enhance stability. Examples include ubiquitination, sulfonation, functionalizing with T4 lysozyme, and fusing with elastin-like protein. Figure created with Biorender.com.

Another method to improve the protection of GFs is to engineer the GFs themselves for enhanced stability. The examples include (1) modifying the ubiquitination site and the addition of (2) a heparin-mimicking domain, (3) T4 lysozyme, and (4) elastin-like protein (EPL) domains. Modifying the amino acid at the site of ubiquitination is one common technique to engineer GFs for increased stability. For example, degradation of BMP-2 occurs through a ubiquitin-mediated mechanism; thus, Khattar et al. mutated individual lysine residues within BMP-2, where ubiquitination mainly occurred. This study showed that substitutions of four lysine residues within the pro-BMP-2 region and three in the mature region increased BMP-2 stability and extracellular secretion [24]. Increasing the stability of GFs by adding a stabilizing domain can also be a solution. Nguyen et al. conjugated basic fibroblast growth factor (bFGF) covalently with a heparin-mimicking polymer, a copolymer consisting of styrene sulfonate units and methyl methacrylate units bearing PEG side chains. Their results showed that bFGF retained bioactivity after the synthesis and was stable under various environmentally and therapeutically relevant stressors, such as heat, mild and harsh acidic conditions, storage, and proteolytic degradation, unlike native bFGF [25]. A recent study fused BMP-2 with T4 lysozyme, a small (164 amino acids) and highly soluble protein, to increase the solubility of BMP-2 and successfully extended its half-life threefold [26]. Elastin-like proteins (ELP) can also prolong the half-life time of GFs. By tuning the ELP transition temperature, GF–ELP fusions can be designed to aggregate and form a micelle structure that protects therapeutic GFs [27]. These methods can dramatically increase the lifetime of therapeutic proteins, contributing to the advancement of therapeutic protein delivery. The strategies used to protect GFs from degradation are summarized in Figure 1.

## 3. Targeted Delivery of Growth Factors

The second critical aspect to consider while developing GF therapies is the delivery of the GF to a desired location. One of the most significant clinical problems in using BMP-2 is ectopic bone formation, i.e., mineralization in undesired tissue regions. Ectopic bone formation results from delivery of BMP-2 not restricted to the target location or weak retention of the BMP-2 to the scaffold when administered locally. In order to prevent GFs from traveling to undesired locations, two strategies can be applied: conjugating GFs with a bone/cartilage targeting motif to actively deliver them to skeletal tissues or immobilizing GFs to a scaffold/biomaterial. In this context, the term “targeted delivery” refers to the active delivery of GFs to a specific location (localized delivery).

### 3.1. Active Delivery with Bone/Cartilage Targeting Motifs

This section introduces common targeting motifs to deliver therapeutic GFs to bone/cartilage actively. We will review the use of small molecule, short peptide sequences, aptamers, and antibody fragments as targeting motifs.

To deliver therapeutic GFs to bone and cartilage, targeting motifs can be conjugated with GFs, such as bisphosphonates (BPs), which inhibit bone digestion by inducing apoptosis of osteoclasts and have a high affinity for bone minerals [28]. Gitten et al. demonstrated that BP-conjugated model proteins bind more strongly to hydroxyapatite than model proteins without BP [29]. Other therapeutic protein or peptides, including osteoprotegerin (OPG), salmon calcitonin, and superoxide dismutase (SOD), can also be conjugated with BP for targeted delivery [30,31,32]. OPG inhibits excessive osteoclastogenesis, which preserves bone mass and helps bone regeneration. Doschak et al. conjugated BPs with OPG and achieved a four-fold higher bone targeting ability compared to the OPG-only control in vivo [30]. Salmon calcitonin is known as a therapeutic polypeptide used for OP. A four-fold increase in targeting to bone mineral components was observed by BP conjugation of calcitonin in vitro [32]. SOD inhibits tumor growth and metastasis [33], and Katsumi et al. conjugated it with polyethylene glycol (PEG) and nitrogen-containing BPs or alendronate (ALN) to achieve targeted delivery. Although systemically administered SOD was not delivered to the bone, ALN–PEG–SOD achieved a 36% delivery rate in vivo [31]. Tetracycline is another small molecule with a high affinity for bone, and it has been shown to have potential applications in bone targeting beyond its use as an antibiotic against bacterial infections [34]. Xie et al. conjugated tetracycline with simvastatin encapsulated in PLGA micelles to achieve targeted delivery of simvastatin to bone [35]. Simvastatin is a small molecule known to stimulate bone formation, increase cartilage thickness, and reduce inflammation at surgical sites.

In addition to small molecules, short peptide sequences are also capable of exhibiting a strong affinity for bone, cartilage, and tendon, making them useful as targeting motifs. One example is the “CAR” peptide sequence, starting from cysteine, alanine, and arginine, which possesses a heparin-binding motif that accumulates in skin and tendon. While Järvinen et al. demonstrated the accumulation of a CAR peptide (CARSKNKDC) in skin and tendon, indicating its potential as a targeting therapy of tendon regeneration, the specificity of the binding of CAR peptide is not high, as it also targets endothelial cells [36,37]. On the other hand, seven aspartic acid repetitions (DDDDDDDC) were found to bind to hydroxyapatite and mineralized matrix, making them effective bone targeting peptides [38]. Using this targeting motif, Sun et al. conjugated the aspartic acid repetitions with P24 (BMP-2 related proteins) to achieve targeted delivery [39]. To achieve cell-specific targeting, phage display methods have been employed in recent studies, such as identifying the “SDSSD” peptide that selectively binds to osteoblasts and the “EPLQLKM” (E7) sequence that specifically binds to MSCs [40,41]. These targeting motifs have been utilized to deliver various therapeutic agents, such as kartogenin for chondrogenic and chondroprotective effects and mi-RNA 140 for its protective role in chondrocytes [41,42,43]. As an application, these targeting sequences can be fused with exosome-enriched proteins so that the sequence would be incorporated in the exosome to create chondrocyte targeting exosomes. Liang et al. utilized chondrocyte targeting exosomes to deliver mi-RNA 140, indicating the potential use of exosomes as a vehicle with an active targeting motif [44]. Newman et al. have summarized several other peptide–nanoparticle conjugations for bone targeting [45].

Aptamers have also been applied as targeting motifs for bone and cartilage. Aptamers are short, single-stranded DNA or RNA, which is developed with high affinity and specificity to interact with selected biological targets [46]. Liang et al. designed an osteoblast-specific aptamer-decorated liposome to deliver siRNA to bone and promote osteoblast function [47]. Aptamers targeting MSCs [48,49], osteoprogenitor cells [50], and type 1 collagen [51] have also been designed and can be used as a targeting motif for protein delivery.

The incorporation of a cell membrane containing bone/cartilage targeting motifs can facilitate active targeting towards the desired tissue, as demonstrated in a study by Park et al., in which genetically modified myeloid cells expressing very late antigen–4 (VLA-4) were used to target inflamed endothelial cells and improve drug delivery efficacy in vivo [52]. They decorated a dexamethasone-encapsulated PLGA with this cell membrane to enable targeted drug delivery, improving the delivery of dexamethasone to inflamed lungs and demonstrating significant therapeutic efficacy in vivo. Cell membrane coating also has the advantage of modulating osteoimmunology favorable for regeneration. A mesenchymal stem cell coating on a bio-scaffold promoted macrophage polarization toward a regenerative phenotype, induced CD8+ T cell apoptosis, and enhanced regulatory T-cell differentiation, ultimately promoting bone regeneration [53].

The utilization of the collagen-binding domain (CBD) represents another effective strategy for achieving targeted delivery of growth factors (GFs). In the past few decades, the CBDs of various collagenases or adhesins have been extensively studied, and fusing these CBDs with therapeutic GFs has proven to enhance bone regeneration. For instance, the CBD of Clostridium histolyticum collagenase was fused to bFGF, resulting in improved bone mineral density and callus volume in a mouse fracture model [54]. Additionally, BMPs, TGFb, PDGF, and PTH have been conjugated to CBD, demonstrating enhanced osteoconductivity in several studies that were comprehensively summarized by Addi et al. [55].

The emerging trend in the development of targeting motifs involves the use of single-chain human antibody fragments (scFv) to precisely guide therapeutic agents to a specific location. Lui et al. employed yeast display technology to create an scFv that targets matrilin-3, an extracellular matrix protein that is specifically expressed in cartilage tissue, and conjugated it with insulin-like growth factor 1 (IGF-1). Their findings indicated a reduced off-target effect on non-targeted tissues, such as the kidney, and a significant increase in growth plate height compared to IGF-1 delivery alone [56]. In a similar study, Ferrari et al. developed a bispecific antibody consisting of scFv-anti-TNFα (adalimumab) and (scFv)-A7 antibodies, which bind strongly to human arthritic synovium, resulting in adalimumab localization to xenografted human synovium in SCID mice, demonstrating the potential of bispecific antibody therapy for rheumatoid arthritis [57]. The bone/cartilage targeting motif with therapeutic GF conjugation mentioned in this section is summarized in the Table 1.

### 3.2. Enhancing Growth Factor Retention in Delivery Scaffolds

Another method to improve GF delivery is to increase the interaction between the GFs and the scaffold/hydrogel so that the GFs do not travel far from the targeted location. In addition to facilitating targeted delivery, this strategy also prevents GFs from acting on nonspecific sites and causing unexpected side effects. One common strategy is to functionalize the high affinity between heparin and GFs [58]. Heparin is a sulfated glycosaminoglycan and has an electrostatic affinity to various kinds of GFs, including bFGF [59] and BMP-2 [60]. In addition, heparin stabilizes GFs, allowing simultaneous immobilization, in vivo localization, and half-life prolongation without chemical crosslinking [61]. For example, Hettiaratchi et al. immobilized heparin to a collagen scaffold to achieve spatial localization of BMP-2, successfully reducing the heterotopic bone formation in a rat femoral defect model [60]. Kim et al. immobilized PDGF-BB and BMP-2 on a heparin-coated PCL scaffold to retain GFs at the scaffold [62]. Another affinity-based retention was conducted by polymerized dopamine. Polymerized dopamine tightly binds to scaffolds via covalent and non-covalent interactions, forming an intermediate layer for biomolecules onto the scaffold surface. Zhou et al. conjugated polymerized dopamine with nano-hydroxyapatite/recombinant human-like collagen/poly(lactic acid) (nHA/RHLC/PLA) to obtain a high affinity for P24 (BMP-2 like protein), resulting in a slower release/higher retention [63].

The covalent conjugation of GFs to a scaffold is another technique to immobilize GFs. Covalent immobilization offers several advantages, such as protecting GFs from degradation, slowing the release kinetics, and lowering the required dosage to achieve biological activities [64]. Karfeld-Sulzer et al. covalently conjugated a BMP-2/BMP-7 heterodimer to fibrin hydrogel to achieve a slower localized release of the BMP heterodimer [65]. Fan et al. covalently immobilized TGFb3 to the scaffold to retain TGFb3, improving cartilage regeneration [66]. The release kinetics of covalently attached GFs follows the degradation of biomaterials, often by enzymatic or hydrolytic cleavage of the chemical bond, thus achieving persistent local delivery of GFs [67]. Briquez et al. developed the dual affinity bridge protein to connect a collagen scaffold and BMP-2 to locally retain protein. The result showed that 80% surface coverage was achieved with as little as 50 ng of BMP-2 with the bridge, indicating a potential application for a clinical trial [68]. Ansari et al. utilized a unique method to retain/capture endogenous BMP-2 by immobilizing anti-BMP-2 monoclonal antibodies (mAbs) on a scaffold. This work did not supply exogenous BMP-2, but successfully entrapped endogenous BMP-2 by immobilizing mAbs to a scaffold. Their results showed improved bone formation in the rat calvarial defect model compared to the iso-control [69]. Though GF itself is not covalently linked to a scaffold in this example, this work demonstrated the importance of GF retention to the scaffold to achieve local delivery. Other than the techniques mentioned here, there are many other strategies, including biotin–streptavidin interactions [70] and peptide amphiphiles to enhance the retaining of GFs to a scaffold summarized elsewhere [71,72]. While immobilization of GFs enhances their stability, it is important to note that this process may also alter the functional activity of GFs. All the works introduced in this section are summarized in Table 2

**Figure 2:**
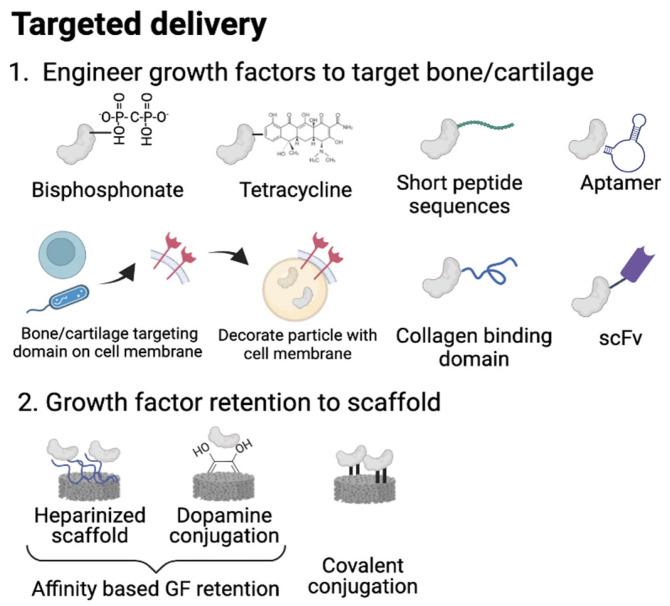
Strategies for targeted growth factor delivery. (**1**) Engineer GFs to target bone/cartilage. Bisphosphonate and tetracycline can be attached to GF to target bone. Short peptide and aptamer can target bone, cartilage, and tendon. A cell membrane containing bone/cartilage targeting motifs can facilitate active targeting towards the desired tissue. A collagen-binding domain and single-chain human antibody fragments can be conjugated with GFs to facilitate active targeting to skeletal tissue. (**2**) Heparinization and dopamine conjugation promote affinity of the scaffold to GFs. Covalent conjugation can be used to retain GFs to the scaffold to avoid for local delivery. Figure created with Biorender.com.

In this section, we have delineated various approaches for delivering therapeutic protein to specific sites within the bone and cartilage microenvironment, with the overarching goal of mitigating potential side effects and optimizing protein delivery efficacy (as depicted in Figure 2). Given that the bone and cartilage regenerative niche encompasses a diverse array of cell types, including skeletal stem cells, osteoblasts, chondrocytes, adipocytes, and immune cells, the impact of therapeutic growth factors extends beyond just the skeletal cells, and can also impact other cell populations. For example, BMP-2 has been shown to promote osteoclast differentiation of mouse bone marrow-derived macrophages, as well as the survival of osteoclasts, and also stimulates adipogenic differentiation of MSCs [73]. With increasingly sophisticated technology enabling the characterization of therapeutic protein effects on multiple cell types, it has become increasingly imperative to achieve targeted delivery of a therapeutic protein in order to maximize its impact on specific cell populations while minimizing unwanted side effects on untargeted cell types [74].

## 4. Controlling GF Release Kinetics

The modulation of growth factor (GF) release kinetics via the implementation of hydrogels/nanocarriers is a pivotal factor in augmenting bone regeneration. The premature and excessive delivery of GFs from hydrogels featuring burst release kinetics can prove deleterious to the regenerative process by conferring a supraphysiological quantity of GFs onto the target cells. Conversely, the optimization of GF dosing for regeneration can be accomplished via the control of release kinetics to minimize off-target delivery. In this section, we will provide a succinct overview of the hydrogels/carriers frequently employed in bone/cartilage regeneration, subsequently delineating the most suitable materials/strategies for short-term or long-term GF release. Lastly, we will explore the strategies for the temporal and spatial control of multiple GFs.

### 4.1. Short-Term Release

If a short-term release of GFs is required, natural biomaterials/carriers, such as alginate [75,76], gelatin [77], liposome [78], and chitosan [79], are adequate choices for a carrier/scaffold. The release kinetics of the therapeutic GFs in these materials ranges from 3 to 14 days and sufficient bone/cartilage regeneration was observed. Synthetic hydrogel can be used to release GFs in the short-term. Olthof et al. encapsulated BMP-2 in a PEG-based hydrogel and controlled its release kinetics for the most optimal bone formation, and found that a scaffold with an initial burst release of BMP-2 induced larger bone formation in a rat subcutaneous implantation model [80]. They implanted 4 ug of BMP-2 in a 24 mm^2^ scaffold, and 75% of the BMP-2 was initially released in 3 days to induce bone formation. Thus, 3 ug of locally administered BMP-2 might be needed to induce bone regeneration effectively. Though this study demonstrates the importance of a local concentration of BMP-2 to trigger bone formation, we also need to consider the side effect of a burst release of BMP-2 that is often seen with the clinical use of INFUSE (clinical BMP-2 product). Olthof et al. did not investigate the BMP-2 effect on the surrounding tissues. A burst release imposes supraphysiological dosing of BMP-2, possibly causing an inflammatory reaction of non-skeletal cells. To avoid such a side effect, skeletal cell type-specific targeted release of BMP-2 might be ideal.

### 4.2. Long-Term Release

While achieving continuous release of growth factors (GFs) for bone and cartilage regeneration is beneficial, it can be challenging to achieve with a longer-term steady release. Synthetic biomaterials are a great choice for a carrier/scaffold to achieve extended long-term release of therapeutic proteins due to their slow biodegradable property. The release kinetics can be prolonged by controlling the polymer’s degradation property, adding a functional group, or coating the carriers. For example, incorporating hydrolytically degradable structures into PEG-based polymers can enable a sustained GF release for over two months [81]. Heparin functionalization can also help achieve long-term release kinetics due to its strong affinity to GFs. Sun et al. conjugated heparin to small intestinal submucosa (SIS) loaded with P28 (BMP-2-like protein), showing 40-days release kinetics and better bone formation in a critical-sized OVX calvarial defect model [82].

Coating carriers with soybean lecithin or dopamine can also prolong GF release, with the former improving the encapsulation efficiency and activity of the GFs [83] and the latter controlling the release kinetics by the thickness of the coating [84]. Another strategy is to encapsulate the target GFs into a nanoparticle and further trap them in a second layer of polymer/hydrogel to prolong the release kinetics. For example, Suliman et al. demonstrated a sophisticated approach by incorporating BMP-2 within PLGA microspheres, which were further encapsulated within a poly(LLA-co-CL) scaffold, leading to an extended release profile of up to 70 days and superior bone formation in a rat critical-size calvarial model, when compared to physiologically adsorbed BMP-2 [85]. Similarly, combining multiple biomaterials to create a multi-layered structure or applying a coating to the carriers can achieve more precise control of the release kinetics [86,87,88].

The successful regeneration of bone and cartilage tissues hinges on the therapeutic growth factors (GFs) and their precise control of the release kinetics. Achieving optimal release kinetics of therapeutic GFs can be accomplished through the selection of appropriate biomaterials, such as natural polymer-based hydrogels for short-term release and synthetic polymer-based hydrogels for long-term release. To further extend the release kinetics, the current approaches involve creating multi-layered structures or applying coatings to carriers to slow down the release. All the studies reviewed in this section are summarized in Table 3.

### 4.3. Synergistic Delivery of Multiple GFs

The delivery of multiple GFs has significant implications for regeneration by activating synergistic pathways. For instance, Li et al.’s research demonstrates that the simultaneous delivery of BMP-2 and BMP-7 enhances the osteogenic ability in MC3T3 osteoblastic cells and improves in vivo bone regeneration [89]. Additionally, co-delivery of BMP-7 and TGFb3 results in enhanced chondrogenesis in vitro [90].

Murphy et al. utilized a different approach by using BMP-2 to amplify skeletal stem cells (SSCs) while reducing VEGF activity through the VEGF receptor (VEGFR) to redirect the fate of SSCs toward chondrocyte/cartilage lineage. By blocking the VEGF signal with VEGFR, endochondral ossification and hypertrophy can be suppressed and the frequency of resting chondrocytes increases, making this a promising strategy to induce cartilage formation [91].

The co-delivery of GFs and small molecules also synergistically enhances regeneration ability, with melatonin known to modulate bone formation and resorption by promoting the differentiation of osteoblast precursors towards osteoblasts, while inhibiting differentiation of osteoclasts [92,93]. Jarrar et al.’s study delivered BMP-2 and melatonin to enhance the osteogenic activity of pre-osteoblastic cells, which was confirmed by in vitro alizarin red and ALP–von Kossa staining, demonstrating enhanced osteogenic activity [94].

### 4.4. Sequential Delivery

The healing of bone and cartilage is a multifaceted process that involves the coordination of various proteins, temporal molecular signaling, and cellular activities across different phases. For instance, BMP-2 is primarily expressed during the early and middle phases of healing, while IGF-1 exhibits peak expression during the early healing phase and weaker expression in the subsequent phases [95]. Proper regulation of the immune response timing is also critical. TNFα, a pro-inflammatory cytokine, plays a complex role during bone healing, with biphasic peaks at 72 h and three weeks after injury [96]. To mimic these natural healing processes, hydrogels/scaffolds are utilized to control the release order of multiple GFs.

Different materials are used to achieve sequential delivery of GFs in bone regeneration therapy. For example, BMP-2 was encapsulated with chitosan particles and dexamethasone in nanofibers to achieve sequential delivery, resulting in the efficient repair of rat calvarial defects [97]. Another study achieved sequential delivery of BMP-2 and alendronate (ALN) by encapsulating ALN in a PLGA microsphere, which was further incorporated in collagen hydroxyapatite. Alendronate is a small molecule used for the treatment of postmenopausal OP due to its strong capacity of promoting osteoblast differentiation and bone formation [98]. The results by Lee et al. showed that sequential delivery improved bone formation in a rat calvarial defect model [99]. Raiche et al. used a layered structure to achieve sequential delivery of BMP-2 and IGF-1, resulting in the largest and earliest elevation of ALP activity and mineralized matrix formation [100]. Similar studies utilizing multiple materials to control the order of release are summarized in Table 4 [101,102,103,104].

### 4.5. Spatial Control

Bone and cartilage are layered structures; therefore, spatial control of releasing the therapeutic GFs becomes very important for proper skeletal healing. Re’em et al. used the high affinity between alginate-sulfate and GFs to control the spatial distribution of bFGF and BMP-4, and the bFGF layer induced chondrogenic differentiation of endogenous cells, while the BMP-4 layer induced endochondral ossification [105]. More recent work has focused on generating gradients of bone and cartilage layers to closely mimic natural bone/cartilage healing. BMP-2 was adsorbed on a PLGA scaffold and TGFb was incorporated in the hyaluronic acid hydrogel; a gradient was created by these two layers. Cartilaginous regions were marked by increased glycosaminoglycan production by the implanted MSCs, and osteogenesis was observed throughout the graft [106].

The 3D printer is another strong tool to spatially control the release of GFs, and its method and examples are well-summarized elsewhere [107]. The advancement of multiple GFs therapy in skeletal regeneration will rely on 3D printer technology. Bone and cartilage are layered 3D structures, making the 3D printer an attractive technology to manufacture implantable constructs. However, not all the materials/GFs reviewed in this section are suitable for 3D-printing due to their chemical and mechanical properties. Some efforts have been made to invent a printable/injectable gel with a controlled release profile. One example is to use a methoxy polyethylene glycol-(polycaprolactone-(N3)) block copolymer (MC-N3). Since MC-N3 exhibits the solution to gelation transition at body temperatures, a subcutaneous injection of the MC-N3 solution into a body results in the rapid formation of a hydrogel depot. The MC-N3 polymer also allows covalent immobilization of BMP-2; thus, injected BMP-2-MC-N3 showed a prolonged release of BMP-2 for 21 days, unlike common injectable hydrogels [108]. The ideal features of biomaterials for bioprinting are bioprintability, high mechanical integrity, insolubility in a medium, biodegradability at a rate appropriate to the regenerating tissue, nontoxicity, non-immunogenicity, and the ability to promote cell adhesion [109]. Development of a bio-printable and GF-laden scaffold will enable the creation of spatially organized structures to regenerate bone and cartilage more precisely. All the studies reviewed in this section are summarized in Table 4.

## 5. Promoting the Long-term Stability of Regenerated Tissues

The long-term stability of implanted scaffolds for maintaining bone/cartilage tissue is another critical factor to consider, particularly in cartilage regeneration, where it is challenging to sustain articular cartilage regenerated by a graft with cells/growth factors (GFs) that are prone to undergo hypertrophy and endochondral ossification in vivo. For instance, while TGFb is a potent cartilage inducer, it can lead to a hypertrophic phenotype of MSCs, as observed in a hydrogel containing MSCs and TGFb3 after 8 weeks of subcutaneous implantation in mice, where calcification was also observed [110,111]. BMP signaling has also been found to promote hypertrophy of chondrocytes [112]. To prevent endochondral ossification, several approaches have been proposed, including inhibiting the Wnt pathway. A study has shown that inhibitors of Wnt signaling pathways are enriched in slow-cycling chondrocytes, which maintain the chondrocyte phenotype without differentiation [113]. Therapeutically, inhibiting β-catenin-mediated canonical Wnt signaling can stimulate chondrocyte hypertrophy, whereas preventing the canonical Wnt pathway can be crucial in maintaining regenerated articular cartilage [114]. A study has also demonstrated that sclerostin, a potent Wnt inhibitor, can restore the chondrogenic phenotype and inhibit endochondral ossification under an IL-1β-induced OA-like environment [115]. The Indian hedgehog (Ihh) pathway is another crucial pathway to consider. In the growth plate, a feedback loop between the Ihh and parathyroid hormone-related protein (PTHrP) plays a significant role in maintaining homeostasis. Chondrocytes produce PTHrP at the end of long bones, which stimulates the proliferation of chondrocytes, preventing them from differentiating further into hypertrophic chondrocytes. However, as cells migrate away from the distal end, PTHrP can no longer reach those cells, and hypertrophic chondrocytes begin synthesizing Ihh. The Ihh signal increases proliferation and accelerates differentiation into prehypertrophic chondrocytes. It also promotes the formation of osteoblasts from adjacent perichondrial cells and completes the feedback loop by promoting PTHrP production at the articular end [116]. Interestingly, in pathological conditions, Ihh expression is upregulated in human OA cartilage, and this upregulation is correlated with OA progression and changes in chondrocyte morphology [117]. Furthermore, Ihh promotes chondrocyte hypertrophy through Wnt/β-catenin activation and bone morphogenetic proteins [118]. Based on these findings, therapeutic delivery of an Ihh antagonist or the addition of PTHrP could be potential solutions to prevent chondrocytes from undergoing hypertrophy [119,120]. In fact, co-delivery of PTHrP with TGFb3 in alginate microspheres results in reduced calcification of long-term cultured MSCs [121].

In addition to targeting the Wnt or Ihh pathway, cartilage regeneration can also be achieved by suppressing osteogenesis with an anti-osteogenic reagent, such as fulvestrant (estrogen receptor antagonist). Hsiao et al. implanted vascularized periosteum together with fulvestrant in critical-size cartilage defects in rabbits to suppress bone formation [121]. While it successfully suppressed the endochondral ossification rate, it also reduced the cartilage regeneration rate. Liu et al. proposed using Matrilin-3 (MATN3), a non-collagenous, cartilage-specific ECM protein, to inhibit endochondral ossification [122]. Regenerated cartilage surrounded by an environment similar to its native setting is likely to maintain its properties. In fact, the subcutaneous implantation of chondrogenic cell/scaffold constructs in a nude mouse model showed that pretreatment with MATN3 was able to maintain chondrogenesis and prevent hypertrophy and endochondral ossification in vivo.

## 6. Osteoimmunomodulatory (OIM) Effects

When developing GF-based regenerative therapy, it is essential to consider osteoimmunology, which focuses on the crosstalk between immune and skeletal cells to create a favorable environment for bone/cartilage regeneration. The modulation of osteoimmunology by both the therapeutic protein and its carrier (biomaterials/hydrogels) is crucial. For example, BMP-2 and TGFb have been found to act on macrophages, leading to diminished expression of M1 phenotypic markers and indicating a positive immunoregulatory role of these growth factors [123,124]. Similarly, IGF-1 and IGF-2 can induce RANKL-independent osteoclastogenesis, contributing to the homeostasis of bone remodeling [125]. Thus, it is evident that therapeutic GFs affect not only osteogenic lineage cells, but also immune cells, making it important to consider their effect on the skeletal cell niche in the development of GF-mediated bone/cartilage regeneration.

Ideally, biomaterials and hydrogels should be designed to synergistically stimulate both immune and skeletal cells towards successful bone regeneration, while avoiding detrimental inflammatory pathways that may lead to bone resorption. Fasolino et al. demonstrated that chitosan-based scaffolds inhibited the production of pro-inflammatory cytokines (IL-1 and IL-6) when osteoblasts and J774A.1 macrophages were co-cultured with LPS stimulation to mimic an inflamed bone environment, indicating their potential to promote bone regeneration under inflammatory conditions [126]. In addition, mesoporous silica rods with cone-shaped pores displayed osteoimmunomodulatory capabilities and reduced the macrophage inflammatory response, potentially creating a microenvironment that favors bone regeneration [127]. Recent research has found that blood clots can also serve as a biomaterial to modulate the OIM environment, attracting M1 pro-inflammatory macrophages during early repair stages, and M2 anti-inflammatory macrophages during later stages of bone remodeling [128]. The surface topology, wettability, and charge of biomaterials also affect osteoimmunology, as detailed elsewhere [129]. The effects of biomaterials on monocyte and T cells are not negligible. Bordoni et al. used graphene oxide with CaP to activate monocytes, resulting in the up-regulation of osteoinductive factor that stimulates bone formation in mice [130]. T cells are also stimulated by biomaterials. For example, titanium dioxide nanorods with a high aspect ratio stimulate T cells, leading to a significant release of FGF-2 and promoting the proliferation of bone marrow-derived stem cells [131].

A thorough understanding of the mechanism underlying the OIM behavior between skeletal cells and immune cells is still a matter of active research. Although the current literature largely supports the idea that M2 macrophages enhance osteogenic differentiation, recent studies have shown conflicting results, likely due to the heterogeneity of the evaluated cells [132]. Various approaches and criteria for isolating, detecting, and evaluating the functional activity of bone progenitors or MSCs have been used, leading to a masking of the true regenerative biology [133]. Therefore, it is crucial to evaluate the OIM effects of scaffolds, hydrogels, and GFs on bone progenitors using well-defined populations. The development of technology allowing for the isolation of a more pure stem cell population, such as bonified skeletal stem cells (SSC) in both mouse [134] and human [135], and the mapping of their lineage commitment towards specific cartilagenic and osteogenic progenitors is a significant step forward. With the aid of single-cell isolation techniques, subpopulations within the prospectively isolated SSC can be further elucidated, leading to the potential to establish a hierarchy tree similar to that of the haemopoietic stem cell. Ultimately, better characterization of the SSC hierarchy will enable a more precise understanding of the effect of various proteins on each cellular sub-population and vice versa, leading to more finely-tuned tissue engineering [136].

## 7. Conclusions and Remarks for Future Research

**Figure 3:**
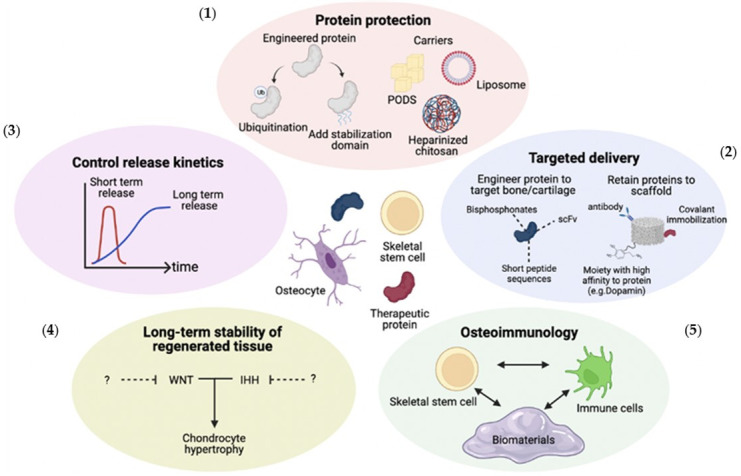
Five important considerations in developing growth factor therapies for bone and cartilage regeneration. For bone and cartilage regenerative therapy, (**1**) protection from physical and enzymatic degradation, (**2**) targeted delivery, (**3**) controlled release kinetics, (**4**) long-term stability of regenerated tissue, and (**5**) the creation of a favorable osteoimmunomodulatory environment are indispensable aspects to consider. Figure created with Biorender.com.

The previous reviews on therapeutic GFs for bone and cartilage regeneration focused on the functions of each of the GFs or engineering the releasing system for controlled delivery [137,138]. In addition to these important topics, our review addresses indispensable aspects of delivering therapeutic GFs, such as the long-term stability of regenerated tissue and osteoimmunology. Our comprehensive review highlights five critical aspects of delivering therapeutic GFs for effective bone and cartilage regeneration, emphasizing the need for protection from physical and enzymatic degradation, targeted delivery, controlled release kinetics, long-term stability of regenerated tissue, and the creation of a favorable osteoimmunomodulatory environment (Figure 3). As our understanding advances, new opportunities are revealed for optimizing skeletal regeneration through the targeted and selective application of therapeutic GFs. Increasingly, progress has been made in developing new platforms using combinations of biomaterials/hydrogels to achieve controlled release of multiple GFs, and this strategy will no doubt be crucial for advancing therapeutic GF delivery in the future.

As the field of bone/cartilage regeneration continues to progress, one emerging challenge is to address the effects of aging. Multiple studies have demonstrated that the ability of older individuals to heal fractures is not as robust as younger individuals [139,140]. To overcome this challenge, we believe that combinatorial GF therapy may offer a potential solution. For instance, a recent study has shown that combining BMP-2 with a colony-stimulating factor 1 (CSF1) antagonist can restore the regenerative capacity of aged mice to that of their youthful counterparts [141]. In order to further optimize these therapies, it will be essential to gain a deeper understanding of the genomic and proteomic underpinnings of skeletal regeneration and aging. By doing so, we can identify and refine combinatorial GF therapies that have the potential to rejuvenate the skeletal system. These therapies can be further improved through the use of controlled release technologies, as discussed in this review.

## Figures and Tables

**Table 1:** Bone/cartilage targeting moiety bound to a therapeutic protein.

Bone/Cartilage Targeting Motif	Structure/Sequence	Target Tissue	Conjugated Protein/Nanoparticles	Result	Ref.
Bisphosphonates(BPs)	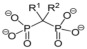 R = single atoms, alkyl chains, amino group etc.	Bone	Osteoprotegerin (OPG)	Four folds increase in targeting bone (tibia) in comparison to OPG only group.	[30]
			Superoxide dismutase (SOD)	BP conjugation achieved 36% of delivery rate while SOD alone showed no accumulation to bone.	[31]
			Salmon calcitonin	4 folds increase in targeting bone mineral component compared to GF only control.	[32]
Tetracycline	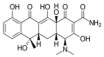		Simvastatin	Preferential accumulation of simvastatin in bone tissue was observed in comparison to simvastatin only control.	[34]
Peptide sequences	CARSKNKDC	Tendon	N/A	CAR sequence was accumulated at tendon and skin *in vitro* and *in vivo*.	[36]
	DDDDDDDC (Poly-Asp)	Bone	P28 (BMP2 related peptide)	Conjugation of poly-asp with P28 to achieve targeted delivery.	[38,39]
	SDSSD	Osteoblast	anti-miR-214 in polyurethane nanomicelles	SDSSD peptide selectively bound to osteoblast via periostin *in vitro*, improving the delivery efficiency *in vivo* with mice osteoporosis model.	[40]
	EPLQLKM	Cartilage	Kartogenin	MSC-targeting sequence (EPLQLKM) improved the efficiency of KGN delivery to MSCs *in vitro* and enhanced cartilage regeneration *in vivo.*	[41]
	DWRVIIPPRPSA		mi-RNA 140	Chondrocyte targeting sequence was encoded after exosome enriched protein, allowing targeted delivery of drug in exosome.	[43]
Aptamer		Bone	siRNA	Osteoblast-specific aptamer-decorated liposome was used to deliver siRNA to bone and promoted osteoblast function.	[47]
Antibody	scFv	Cartilage	IGF1	Conjugation of IGF1 with scFV targeting matrilin-3 showed enhanced IGF1 accumulation at cartilage and reduced off-target delivery at other organ.	[56]
		Synovium	scFv-anti-TNFa (Adalimumab) and scFv-A7	Bispecific antibody; one targeting to TNFa to suppress inflammation and other end targeting to synovium (scFV-A7). Results showed successful accumulation at xenografted human synovium in mice.	[57]

**Table 2:** Method to retain the proteins at the scaffold.

Protein	Scaffold	Method	Result	Ref.
BMP2	Collagen	Affinity	Heparin was immobilized to collagen scaffold to achieve the spatial localization of BMP2, successfully reducing the heterotopic bone formation.	[60]
PDGF-BB BMP2	PCL		GFs were immobilized on heparin coated PCL scaffold. Continuous release of initial loading amount after 5 weeks without an initial burst were observed, leading to better tendon regeneration.	[62]
P24	nHA/RHLC/PLA		Polydopamin was coated on nHA/RHLC/PLA to have an affinity to P24, resulting slower release/high retention.	[63]
BMP2 BMP7	Fibrin	Covalent	Covalent conjugation allowed slower release kinetics *in vitro*. Enhanced bone regeneration was observed in critical size calvarial defects model with rat.	[65]
TGFβ3	PLGA-GCH		Prolonged release of TGFβ and improved cartilage regeneration were observed.	[66]
BMP2	Collagen	Engineered bridge	Dual affinity bridge protein connected collagen scaffold and BMP2. Lower BMP2 dosage was required to induce bone formation *in vivo*.	[68]
	Collagen/alginate/Titanium	Antibody	BMP2 mAbs was immobilized to scaffold to capture endogenous BMP-2 for bone regeneration, improving bone formation in rat calvarial defects model.	[69]
BMP2 FGF2	Gelatin	Biotin-avidin	Biotinylated BMP2 and FGF2 were bound to avidin functionalized nanofiber, showing controlled release of BMP2 and FGF2.	[70]

**Table 3:** Time-controlled release strategies for bone/cartilage regeneration.

	Materials	Modification	Protein	Target	Release Duration	Result	Ref.
Natural	Alginate sulfate	Sulfation	bFGF	Vascular	5 days	Slower release with sulfate-conjugated alginate. Sulfated alginate released 50% of bFGF by day 5 while control alginate released 50% of bFGF at day 0.	[75]
			TGFb	Cartilage	7 days	The sulfate group exhibited an affinity for TGFb, resulting in a slower release rate compared to non-sulfated alginate.	[76]
	Gelatin-PCL	N/A	BMP2	Bone	10–45 days	Gelatin/heparin gel enhanced cells viability and PCL enhanced mechanical property. Release kinetics was controllable by combining gelatin and PCL.	[77]
	Liposome				100 hours	Achieved steady release of rh-BMP2 for 100 hours *in vitro*.	[78]
	Chitosan	Thiolation	BMP2	Bone	14 days	The thiolate modification contributed to the upregulation of ALP activity and better bone regeneration by prolonging the release of BMP2.	[79]
Synthetic	PEG-based hydrogel	PLGA microparticle	BMP2	Bone	3 days	BMP-2 was encapsulated within PLGA microparticles, which were further enclosed within a PEG-based hydrogel. Initially, 75% of the BMP-2 was released within 3 days.	[80]
		N/A	bFGF	Cartilage	60 days (BSA) 35 days (FGF)	Hydrolytically degradable structures increased the hydrogel swelling ratio and mesh size, enabling sustained protein release over 2 months.	[81]
	P34HB nanoparticles	Soybean coating	BMP7	Bone	20 days	Soybean coating on nanoparticle significantly slowed down BMP7 release kinetics.	[83]
	Mesoporous silica in Hydrogel	Dopamine coating	TGFb3	Cartilage	75 days	TGFb3 was loaded in mesoporous silica coated with DOPA. Thicker DOPA coating achieved slower release and achieved 75 days release duration in vitro.	[84]
	PLGA particle in poly(LLA-co-CL)	N/A	BMP2	Bone	70 days	BMP2 was incorporated in PLGA microsphere, which were further encapsulated in scaffold. Result showed better bone formation in rat calvarial model.	[85]
	Mesoporous silica in PLGA		BMP2	Bone	40 days	BMP2 was incorporated in Mesoporous silica, then mixed with PLGA to create microsphere. *In vitro* functional assay showed improved bone formation.	[86]
	MBG/SIS scaffold	Heparin	P28	Bone	40 days	BMP2 was incorporated in MBG, which were further encapsulated in SIS. Enhanced bone regeneration was observed in rat calvarial defect model.	[87]
Hybrid	hyaluronate/type I collagen/fibrin composite containing PVA nanofibers enriched with liposomes	N/A	bFGF Insulin	Cartilage	19 days	Achieved steady release of both bFGF and insulin for 19 days in vitro. Nanofiber provided mechanical stiffness and elasticity closer to native cartilage. In vivo mini-pig experiment demonstrated cartilage regeneration.	[88]

**Table 4:** Multiple protein release strategies for bone/cartilage regeneration.

System	Protein	Target	Mechanism	Release Kinetics/Spatial Release Strategy	Result	Ref.
Synergistic	BMP7 & BMP2	Bone	BMP7 and BMP2 were loaded in PELA microparticle.	BMP7 and BMP2 showed steady release for 42 days *in vitro*.	*In vivo* rat femoral defect model demonstrated improved bone regeneration.	[89]
	BMP7 & TGFb3	Cartilage	BMP7 and TGFb were loaded in PLGA microsphere.	BMP7 and TGFb showed steady release for 30 days *in vitro.*	Synergistic effect of chondrogenic promotion *in vitro*.	[90]
	BMP2 & VEGFR	Cartilage	BMP2 and VEGFR were loaded in PEG based hydrogel.	BMP2 promoted osteogenic differentiation of SSCs, which was further directed to chondrocyte with VEGFR.	Implantation of hydrogel containing BMP2 and VEGFR at femoral defect promoted cartilage formation.	[91]
	BMP2 & Melatonin	Bone	BMP2 and Melatonin were loaded in PLGA microparticle, which is further encapsulated in Chitosan-Hap scaffold.	BMP2 and melatonin showed steady release for 20 days *in vitro*.	Improved osteogenic ability was confirmed by alizarin red and ALP-von Kossa staining using MC3T3-E1 cells.	[94]
Sequential delivery	BMP2 & Dex	Bone	BMP2 is encapsulated in chitosan particle, which is further incorporated in PCE nanofiber with DEX.	Dex exhibited a burst release during the first 5 days, whereas BMP2 displayed a consistent release over 35 days.	Dual delivery demonstrated a better bone regeneration in rat calvarial bone defect.	[97]
	BMP2 & ALN	Bone	ALN is encapsulated in PLGA microsphere, which is further incorporated in collagen hydroxyapatite.	The release profile of BMP2 exhibited a burst kinetics for the first 5 days, whereas ALN demonstrated a delayed release between 2 to 6 weeks.	Dual delivery demonstrated a better bone regeneration in rat calvarial bone defect.	[98]
	BMP2 & IGF	Bone	BMP2 was encapsulated in the 1st gelatin layer and BMP2 and IGF were loaded in the 2nd gelatin layer.	BMP2 in 1st layer was released in 2 days and 2nd layer in 6 days.	Increased AP activity and matrix calcium content compared to control.	[100]
	SDF1 & BMP2	Bone	BMP2 is encapsulated in silk fibroin particle, which is further incorporated in Hap scaffold with SDF1.	SDF1 demonstrated a burst release for first 5 days while BMP2 showed steady release for 35 days.	Dual delivery showed a better bone regeneration in rat calvarial bone defect.	[101]
	TGFb & BMP2	Cartilage	TGFb is encapsulated in gelatin microparticle and BMP2 in mineral-coated hydroxyapatite microparticles.	TGFb displayed an initial burst release for 10 days and sustained BMP2 release for 60 days.	Dual delivery resulted in an enhanced GAG and Col2 expression, as demonstrated by immunostaining.	[102]
	IGF1& TGFb1	Cartilage	IGF1 is incorporated in gelatin microparticle, which is encapsulated in OPF with TGFb.	An initial burst release of TGFb was observed, followed by a slower release of IGF1.	The release kinetics were able to be adjusted by modifying the crosslinking amount.	[103]
	IL-8 & BMP2	Bone	BMP2 is incorporated in mesoporous bioactive glass (MBG) which is coated by PEG with IL-8.	Initial burst release of IL-8 for 1 day and steady release of BMP2 for 7 days.	The recruitment of stem cells by IL-8 and the promotion of osteogenesis by BMP2 resulted in enhanced bone regeneration.	[104]
Spatial control	bFGF & BMP4	Bone& Cartilage	Use high affinity between sulfate and proteins to control spatial distribution.	Two layered alginate-sulfate: One layer with bFGF, another one with BMP.	bFGF induced chondrogenic differentiation. BMP4 induced endochondral ossification of endogenous cells.	[105]
	BMP2 & TGFb	Bone& Cartilage	hyaluronic acid hydrogel was filled in porous PLGA scaffold.	BMP2 adsorbed to PLGA scaffold and TGFb incorporated in the hydrogel. Gradient was created by these 2 layers.	Cartilaginous regions were marked by increased GAG production, and osteogenesis was seen in the graft.	[106]

## Data Availability

Not applicable.
